# Nutritional Knowledge, Physical Activity, Mood, Body Satisfaction, and Life Satisfaction in Vegetarians and Nonvegetarians

**DOI:** 10.1155/jnme/1907455

**Published:** 2025-05-03

**Authors:** Diana M. Quitian Puentes, Mariotty Severiche Ortega, Percy G. Ruiz-Mamani, Jacksaint Saintila, Salomón Huancahuire-Vega

**Affiliations:** ^1^Unidad de Ciencias de la Salud, Escuela de Posgrado, Universidad Peruana Unión, Lima, Peru; ^2^Facultad de Derecho y Ciencias Empresariales, Universidad Privada San Juan Bautista, Lima, Peru; ^3^Research Group for Nutrition and Healthy Behaviors, School of Medicine, Universidad Señor de Sipán, Chiclayo, Peru; ^4^Departamento de Ciencias Básicas, Escuela de Medicina Humana, Facultad de Ciencias de la Salud, Universidad Peruana Unión, Lima, Peru

**Keywords:** adult, mood, physical activity, vegetarians

## Abstract

**Background:** Knowledge of nutritional aspects, the practice of physical activity, body satisfaction, and explanation of mood is a topic of great relevance in the field of nutrition, as it allows us to understand in a more exhaustive way the implications of the eating pattern on satisfaction with people's lives. The objective of this study was to determine the relationship between nutritional knowledge, dietary pattern, physical activity, mood, body satisfaction, and life satisfaction in vegetarian and nonvegetarian Colombian adults.

**Materials:** Study with a quantitative, observational, multivariate, correlational, and cross-sectional approach. The variables dietary pattern, nutritional knowledge, physical activity, mood, body satisfaction, and life satisfaction were analyzed. The sample (*N* = 478) included the participation of vegetarians (*N* = 157) and nonvegetarians (*N* = 321); the selection of the sample was carried out by nonprobabilistic accidental sampling. The data were collected through an online questionnaire, processed using SPSS version 26 and R version 4.4.2, and analyzed with descriptive statistics. The effect size was calculated based on mean differences, and Spearman's Rho correlation test was applied, considering a significance level of 0.05.

**Result:** In vegetarians, physical activity showed a positive correlation with mood (*r* = 0.210, *p* < 0.01). Body dissatisfaction demonstrated a significant negative correlation with life satisfaction (*r* = −0.26, *p* < 0.01) and mood (*r* = −0.28, *p* < 0.01). Body dissatisfaction showed a significant positive correlation with BMI (*r* = 0.30, *p* < 0.01). Life satisfaction was positively correlated with mood (*r* = 0.54, *p* < 0.01). Nutritional knowledge exhibited a significant negative correlation with BMI (*r* = −0.17, *p* < 0.05). Mood also showed a significant positive correlation with BMI (*r* = 0.16, *p* < 0.05). Among nonvegetarians, body dissatisfaction was negatively and significantly correlated with life satisfaction (*r* = −0.29, *p* < 0.01) and positively correlated with BMI (*r* = 0.29, *p* < 0.01). Life satisfaction was significantly positively correlated with mood (*r* = 0.42, *p* < 0.01) and negatively correlated with BMI (*r* = −0.12, *p* < 0.05). Nutritional knowledge showed a positive correlation with life satisfaction (*r* = 0.14, *p* < 0.05), while mood was negatively correlated with body dissatisfaction (*r* = −0.36, *p* < 0.01).

**Conclusion:** These results suggest the importance of nutritional education and its need to adopt a comprehensive approach that includes dietary aspects and considers the relationship between diet, physical activity, and emotional well-being to promote healthy habits and a better quality of life.

## 1. Introduction

In a report from the Food and Agriculture Organization of the United Nations (FAO), it has been reported that the diets and eating habits in Latin America and the Caribbean have changed in the last 3 decades [1]. The above is consistent with the results of the National Survey of the Nutritional Situation in Colombia (ENSIN) 2015 [2], which has highlighted the problem of the food and nutritional transition associated with the increase in the consumption of unhealthy foods, such as soft drinks and sugary soft drinks, fast food rich in free sugars and saturated fats, as well as the general imbalance in the consumption of fats, carbohydrates, and proteins [3, 4]. These aspects, in turn, can impact nutritional status, causing malnutrition, overweight, and obesity, as well as problems related to mood, body dissatisfaction, and dissatisfaction with life [5, 6]. Furthermore, previous research has suggested that vegetarian diets are often linked to broader health-promoting habits. Studies have indicated that vegetarians, compared to nonvegetarians, may exhibit higher levels of nutritional awareness and engage in healthier eating behaviors [7]. In addition, there is evidence that vegetarians may also report better psychological well-being, such as improved mood and higher life satisfaction [8–11]. This is likely because of the greater intentionality involved in adhering to a plant-based diet, which often requires a conscious focus on overall health and nutrition.

On the other hand, lack of physical activity is considered one of the main predictors of chronic conditions, such as excess body weight (overweight and obesity), type 2 diabetes mellitus, cancer, cardiovascular diseases, and high blood pressure, among others [12]. Also, people who do not do physical activity have a 20%–30% higher risk of death compared to those who do physical activity [13]. Globally, it is estimated that one in four adults does not meet the physical activity levels recommended by the World Health Organization [13]. On the other hand, physical activity positively affects people's health, demonstrating a preventive impact on diseases [14]. Similarly, findings from previous studies have shown that it can positively affect mood [15]. Also, another study reported that physical activity can help people have a positive self-concept and promote psychological well-being by improving physical perceptions and body satisfaction [16]. Furthermore, it has been shown that people who were more satisfied with their body size were more likely to engage in regular physical activity than those who were less satisfied [17]. Likewise, physical activity has beneficial effects on people's psychological health. In a study that evaluated 2345 healthy adults, physical activity was significantly related to life satisfaction in young adults [18]. Therefore, the promotion of physical activity is necessary to ensure not only physical health but also mental health. Although research comparing physical activity levels between vegetarians and nonvegetarians is less common, there are indications that vegetarians, who tend to prioritize health-conscious behaviors, may also be more physically active. Regular physical activity has been shown to improve body satisfaction and life satisfaction, further promoting a holistic approach to health [15, 16, 18]. These findings suggest that adopting a vegetarian diet may lead to an overall healthier lifestyle.

Lack of knowledge about healthy eating and nutrition is one of the leading causes of inadequate dietary habits in both vegetarians and nonvegetarians [19]. Previous studies have shown that both populations often exhibit poor knowledge about nutrition and dietary recommendations [20]. Therefore, it is necessary to implement nutritional education programs for both population groups. However, vegetarians, in particular, may be at greater risk of nutrient deficiencies if their nutritional knowledge is insufficient, as they must carefully plan their diets to ensure adequate intake of essential nutrients like calcium, iron, zinc, and vitamin B12—nutrients that are more commonly found in animal products [21]. Deficiencies in these nutrients can lead to a range of health issues, including anemia (iron), compromised bone health (calcium), impaired immune function (zinc), and neurological problems (vitamin B12) [22, 23]. Given these risks, it is important to implement targeted nutritional education programs that address the specific dietary needs of vegetarians. Nutritional knowledge has the potential to improve eating habits, as studies have reported that greater nutritional knowledge is associated with lower consumption of fats and sugars and higher intake of fruits and vegetables [24, 25]. However, it is also important to note that some studies suggest that even with sufficient knowledge, this may not always translate into healthier eating behaviors, nor does it necessarily reduce body dissatisfaction [26, 27]. This discrepancy highlights the importance of addressing both knowledge and behavior in nutritional interventions, particularly for vegetarians who may be at higher risk for certain deficiencies.

The comparison between vegetarians and nonvegetarians in this study is essential to understand how different dietary patterns can influence not only nutritional knowledge but also key aspects of well-being such as mood, body satisfaction, and life satisfaction. Vegetarians, because of their specific dietary choices, may have greater nutritional awareness, which could positively affect physical and psychological health. On the other hand, nonvegetarians, while potentially having a more varied intake of nutrients, may experience different levels of body dissatisfaction or mood based on their eating habits. Exploring these differences provides valuable insights into how dietary choices are related to overall health and well-being, particularly in a Colombian context, where dietary behaviors may differ from other populations. This study seeks to fill this gap by comparing these two groups and identifying potential areas for targeted nutritional education and interventions aimed at improving both physical and mental health outcomes.

Understanding the level of nutritional knowledge is very relevant because it would help detect nutritional needs to design and apply specific interventions to the diet of vegetarians and nonvegetarians. Therefore, this study aims to determine the relationship between nutritional knowledge, physical activity, mood, body satisfaction, and life satisfaction among Colombian vegetarian and nonvegetarian adults.

## 2. Materials and Methods

### 2.1. Study Design and Participants

An online cross-sectional study was conducted on 478 Colombians aged between 18 and 60 years, of whom 157 were vegetarians and 321 were nonvegetarians. The sample was selected by nonprobabilistic accidental sampling. All vegetarian participants who reported adherence to the diet for at least 1 year were included. Pregnant or breastfeeding women, individuals with disabilities, depression, diagnosed with chronic diseases, and those residing outside of Colombia were excluded to ensure sample homogeneity and reduce the potential for biases that could affect the study results, as these conditions can significantly alter some of the variables under investigation. The data were collected between March and June 2021.

The instruments were applied through Google Forms, where a survey was conducted: (1) Information about informed consent; participants could accept or decline to answer the survey questions. (2) Sociodemographic data registration form that included age in years (18–25, 26–34, 35–43, and ≥ 44), gender (men and women), occupation, and marital status (single and being in a relationship), city of residence, religion, socioeconomic stratum, and level of education (technical, university, and postgraduate).

The instrument was distributed via email, social networks of vegetarian and nonvegetarian population groups, and other digital media, accompanied by an informative message explaining the form to be completed. This approach enabled efficient collection of data within a shorter timeframe. In addition, this strategy facilitated access to a diverse sample of vegetarian and nonvegetarian adults in Colombia, who voluntarily consented to participate in the study.

### 2.2. Ethical Considerations

Before data collection, informed consent was requested and obtained from all participants. The Research Ethics Committee of the Faculty of Health Sciences of the Universidad Peruana Unión approved the execution of this study (Approval reference number: 2021-CE-FCS-UPeU-00182). Finally, all procedures contributing to the study followed the ethical criteria of the 1975 Declaration of Helsinki, and its later amendments [28].

### 2.3. Data Collection Instruments

  Nutritional knowledge: The short nutritional knowledge scale (CoNKS) was used to measure the levels of nutritional knowledge of the participants [29]. This instrument is composed of three subscales: (1) dietary recommendations, (2) nutrient sources, and (3) dietary choices. In addition, it contains 20 items with a true or false response option. This questionnaire was validated, and acceptable internal consistency values were found, Cronbach's Alpha 0.73.
  Dietary regimen: A consumption frequency questionnaire based on an exchange system was applied, and it has been validated in a previous study [30]. This instrument was used to characterize the dietary regimen of the participants, classifying them as follows according to their responses: nonvegetarians (there are no specific nutritional restrictions about the frequency of consumption of foods, such as meat, fish, dairy products, and derivatives) and vegetarians (lacto-ovo-vegetarians): consumption of milk and derivatives, eggs, and foods of plant origin. Vegans: elimination of all products of animal origin [31].
  Physical activity: The International Physical Activity Questionnaire–Short Form (IPAQ-SF) assessed the participants' physical activity levels. This instrument is considered an appropriate method to evaluate physical activity at a population level and is designed to be self-administered. In addition, it is composed of seven questions that inquire about three specific characteristics: (1) intensity, (2) frequency, and (3) duration of physical activity [32]. The questionnaire was validated in the Colombian population and presented a Cronbach's Alpha of 0.78 [33]. Furthermore, the instrument has been used in other studies in this population [34].
  Body satisfaction: This variable was assessed using the Body Satisfaction Questionnaire (BSQ-8C), a short version of the scale designed to measure body dissatisfaction. The scale includes eight questions with Likert-type response options ranging from 1 to 5. The instrument was validated in the Spanish-speaking population, demonstrating adequate internal consistency with *α* = 0.91 and *ω* = 0.89. [35]. It is considered the most favorable version because of its psychometric properties and sensitivity to change and is recommended for research (clinical and nonclinical) [36]. Although the scale is named the Body Satisfaction Questionnaire, for the purposes of this study, it will be referred to as body dissatisfaction, aligning with what the instrument specifically measures.
  Mood: This is the short version of the mood assessment scale (EVEA). This scale showed high internal consistency indices; this short version does not include the anger-hostility subscale. This version consists of 9 items with a response option scale of 0–10; the original version involves 16 items with a scale type of 11-point Likert [37].
  Satisfaction with life: The satisfaction with life scale (SWLS) comprises five questions with seven-point Likert-type response options. This scale has been validated in the Colombian population with a Cronbach's Alpha of 0.84 [38]. A study carried out with the adult population also reported that the available Spanish translation can be used to measure satisfaction with life in the Colombian population [39].

### 2.4. Statistical Analysis

The statistical software IBM SPSS (Statistical Package for the Social Sciences) version 26 and R program version 4.4.2 were used for data analysis, including data cleaning and debugging. Descriptive statistics were applied to describe the characteristics of the population, categorized by dietary patterns using frequencies and percentages. For the comparative analysis of the variables, the Kolmogorov–Smirnov normality test was applied, rejecting the null hypothesis that the data follow a normal distribution (*p* < 0.05). Despite this, both the Mann–Whitney *U* test and Student's *t*-test were conducted for comparative analyses. No differences were found between the results of the two tests. For the purpose of calculating the effect size, the differences in means were used. The correlations between the study variables were determined using Spearman's Rho test, with a significance level set at 0.05.

## 3. Results

### 3.1. Distribution of Sociodemographic Characteristics Among Vegetarian and Nonvegetarian Participants

The majority of participants in both groups fall within the 26–34 years range (35.8%), with a slightly higher representation of vegetarians in this category. The sample is predominantly composed of women (67.4% overall), with a slightly higher proportion of women among vegetarians (68.2%) compared to nonvegetarians (67.0%). Approximately, 55.6% of the participants are single, with similar proportions in the vegetarian and nonvegetarian groups (53.5% and 56.7%, respectively). In terms of socioeconomic status, 36.6% of the sample belongs to stratum 3, with vegetarians being more represented in the lower strata, particularly in stratum 1 (14.6% among vegetarians compared to 12.5% among nonvegetarians). More than 53.6% of the total sample has a university education, with vegetarians showing a slightly higher proportion of technical studies. Lastly, 38.9% of participants are classified as overweight. Notably, vegetarians have a higher proportion of individuals with a normal BMI (73.9%) and a lower prevalence of overweight (21.7%) compared to nonvegetarians, who show a higher prevalence of overweight (47.4%) (Table 1).

### 3.2. Comparative Analysis of Variables Between Vegetarians and Nonvegetarians

No significant differences were observed between the two groups in relation to physical activity (*t* = 1.03, *p* > 0.05), life satisfaction (*t* = −0.71, *p* > 0.05), and mood (*t* = 1.13, *p* > 0.05). However, a significant difference was found in body dissatisfaction (*t* = −3.07, *p* < 0.01), with nonvegetarians reporting greater dissatisfaction (*M* = 18.64) compared to vegetarians (*M* = 16.27), with a moderate effect size (*d* = 0.30). In addition, significant differences were observed in nutritional knowledge (*t* = 5.51, *p* < 0.001), where vegetarians demonstrated higher knowledge (*M* = 15.13) compared to nonvegetarians (*M* = 13.61), with a large effect size (*d* = 0.55). Finally, body mass index (BMI) was significantly lower in vegetarians (*M* = 23.34) compared to nonvegetarians (*M* = 24.96) (*t* = −4.34, *p* < 0.001), with a moderate effect size (*d* = 0.41), indicating that vegetarians have a lower BMI (Table 2).

### 3.3. Analysis of Correlations Between Variables

Among vegetarians, physical activity is positively correlated with mood (*r* = 0.210, *p* < 0.01). Body dissatisfaction shows a significant negative correlation with life satisfaction (*r* = −0.26, *p* < 0.01) and mood (*r* = −0.28, *p* < 0.01). In addition, body dissatisfaction is significantly positively correlated with BMI (*r* = 0.30, *p* < 0.01). Conversely, life satisfaction is positively correlated with mood (*r* = 0.54, *p* < 0.01). Nutritional knowledge exhibits a significant negative correlation with BMI (*r* = −0.17, *p* < 0.05), suggesting that individuals with greater nutritional knowledge tend to have a lower BMI, though the effect size is small. Lastly, mood shows a positive correlation with BMI (*r* = 0.16, *p* < 0.05), indicating that better mood is slightly associated with higher BMI, although the effect size remains small (Table 3).

Among nonvegetarians, the correlation results indicate that physical activity does not show significant relationships with any of the other variables. However, body dissatisfaction is negatively and significantly correlated with life satisfaction (*r* = −0.29, *p* < 0.01), indicating that higher body dissatisfaction is associated with lower life satisfaction, with a moderate effect size. In addition, body dissatisfaction shows a significant positive correlation with BMI (*r* = 0.29, *p* < 0.01), suggesting that individuals who are more dissatisfied with their bodies tend to have a higher BMI, also with a moderate effect size. In relation to life satisfaction, a significant positive correlation was observed with mood (*r* = 0.42, *p* < 0.01), indicating that higher life satisfaction is associated with better mood, with a moderate effect size. A significant negative correlation was also found with BMI (*r* = −0.12, *p* < 0.05), suggesting that a higher BMI is slightly associated with lower life satisfaction, although the effect size was small (Table 3).

**Figure 1:**
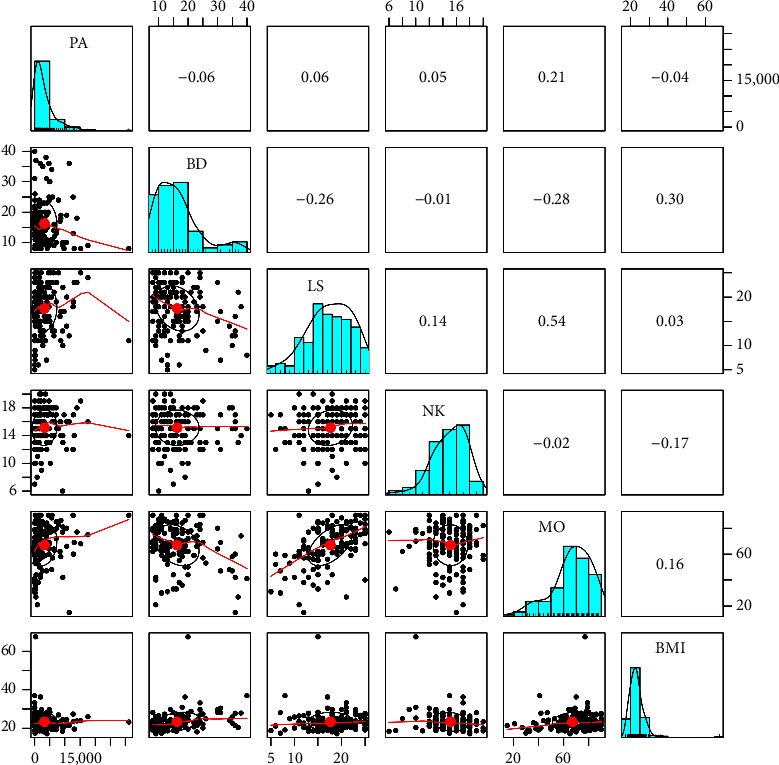
Correlations between variables and scatter plots in vegetarians. Note. PA = physical activity, BD = body dissatisfaction, LS = life satisfaction, NK = nutritional knowledge, MO = mood, BMI = body mass index.

**Figure 2:**
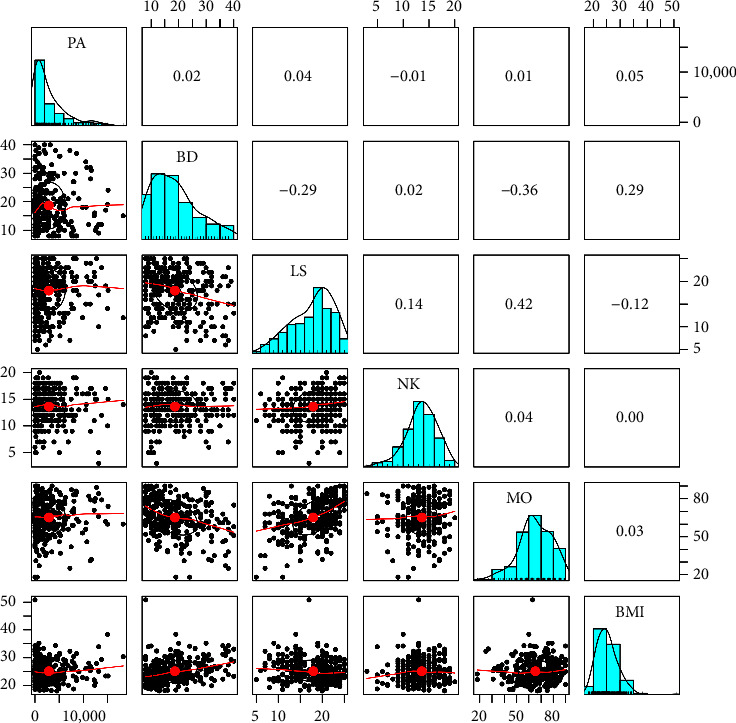
Correlations between variables and scatter plots in nonvegetarians. Note. PA = physical activity, BD = body dissatisfaction, LS = life satisfaction, NK = nutritional knowledge, MO = mood, BMI = body mass index.

On the other hand, nutritional knowledge exhibits a positive correlation with life satisfaction (*r* = 0.14, *p* < 0.05), suggesting that individuals with greater nutritional knowledge tend to be more satisfied with their lives, although the relationship is small. In addition, mood is negatively and moderately correlated with body dissatisfaction (*r* = −0.36, *p* < 0.01), indicating that higher body dissatisfaction is associated with worse mood (Table 3), see also Figures 1 and 2.

## 4. Discussion

This study aimed to determine the relationship between nutritional knowledge, dietary pattern, physical activity, mood, body satisfaction, and life satisfaction in vegetarian and nonvegetarian Colombian adults. In both vegetarians and nonvegetarians, body dissatisfaction was found to have a significant negative correlation with life satisfaction and mood, and a positive correlation with BMI. Life satisfaction was also significantly correlated with mood in both groups. Among vegetarians, mood showed a positive correlation with physical activity and BMI, while BMI showed a negative correlation with nutritional knowledge. Among nonvegetarians, nutritional knowledge was found to exhibit a positive correlation with life satisfaction.

The analysis of dietary patterns in relation to body satisfaction revealed significant differences between vegetarians and nonvegetarians, with nonvegetarians experiencing greater body dissatisfaction. This finding aligns with previous studies suggesting that individuals following vegan or vegetarian diets tend to report higher levels of body satisfaction compared to those on omnivorous diets [40]. In addition, research indicates that individuals who engage in healthy behaviors generally exhibit greater body satisfaction than those with less healthy habits [41]. According to the literature, body satisfaction is greater among those who practice healthy habits. However, other studies have observed that vegetarians tend to show less concern about controlling body weight [42]. Interestingly, while vegetarians in this study may have exhibited less concern about weight control—a finding consistent with other research—75% of them maintained a normal BMI. This suggests that their dietary habits support healthy body weight management without heightened weight-related anxiety. This is significant because body dissatisfaction, influenced by both internal factors and societal pressures to conform to idealized body standards, can lead to unhealthy behaviors, including eating disorders [43]. Therefore, understanding how dietary patterns influence body image is crucial for addressing and potentially mitigating body dissatisfaction and its harmful consequences [44].

On the other hand, this study confirmed that vegetarians' nutritional knowledge is significantly greater than that of nonvegetarians. It is likely that because the diet adopted by vegetarians is associated with healthier habits, these individuals have better nutritional and health knowledge [45]. However, although vegetarians tend to exhibit better health indicators, significant differences are only sometimes observed in terms of levels of nutritional knowledge [46]. Today, nutritional and general health knowledge is more accessible to the population than it was a few years ago, meaning that vegetarians and nonvegetarians have the same learning opportunities. However, having access to knowledge does not guarantee that it will lead to changes in people's lifestyles and nutritional habits. This suggests that while knowledge is an important foundation, other factors, such as motivation, cultural influences, and individual priorities, play a crucial role in transforming knowledge into actionable, healthier lifestyle choices. Therefore, understanding the gap between awareness and actual practice is essential for promoting healthier behaviors, regardless of one's dietary preferences.

This study underscores the significant role that nutritional knowledge plays in promoting positive health behaviors, including overall life satisfaction and BMI. Among vegetarians, a clear link was found between higher nutritional knowledge and BMI, suggesting that individuals with greater nutritional knowledge tend to have a lower BMI [47]. The findings align with other research, which consistently shows that individuals with better nutritional knowledge are more likely to meet health guidelines and improve their overall nutritional habits [47]. Importantly, this highlights the transformative potential of education and knowledge dissemination, as interventions aimed at improving nutritional awareness can act as catalysts for healthier behaviors, reducing the risk of chronic diseases, enhancing weight management, and increasing life satisfaction. Ultimately, the integration of nutritional education with lifestyle interventions represents a powerful approach to fostering long-term health and well-being across diverse populations.

Life satisfaction was also positively correlated with nutritional knowledge. The literature consistently demonstrates a positive relationship between nutritional knowledge and life satisfaction. This relationship is complex and extends beyond the physical health benefits of good nutrition [48]. Another study investigated the impact of a lifestyle educational intervention, including a nutritional education program, on the life satisfaction of overweight or obese patients. Participants in the nutrition education program reported reduced body weight parameters and improved psychological health. In addition, they reported higher levels of life satisfaction, attributing their enhanced well-being to a better understanding of how nutrition affects their health [45]. This relationship is particularly evident in interventions where participants, such as overweight or obese individuals, report improvements in both body weight and psychological health after receiving nutritional education. These findings suggest that empowering individuals with nutritional knowledge can lead to holistic health benefits, fostering both physical wellness and a greater sense of life satisfaction, highlighting its value as a key component in health promotion efforts.

Physical activity has traditionally been associated with numerous physical and mental health benefits [46]. However, emerging research suggests that the relationship between physical activity and mood may be more complex than previously thought. The positive correlation between mood and physical activity was observed only in nonvegetarians. Recent research suggests that physical activity typically enhances mood by reducing symptoms of depression and anxiety across various diet groups, including vegetarians and omnivores. In addition, engaging in physical activity boosts endorphin levels, which improves mood and lowers stress, irrespective of dietary choices [49]. However, unique factors affecting vegetarians might explain the lack of correlation observed. For instance, vegetarians sometimes experience higher levels of psychological stress because of societal pressures and stigmatization, which could overshadow the mood benefits typically gained from physical activity. Furthermore, nutrient deficiencies, such as vitamin B12 or omega-3 fatty acids, which are less abundant in plant-based diets, could negatively impact mood and neurological health, potentially reducing the positive mental effects of physical activity in this group [50].

Interestingly, in this study, it was found that the correlation between body dissatisfaction and life satisfaction is negative in both vegetarians and nonvegetarians. Body dissatisfaction, or dissatisfaction with physical appearance, is a fundamental aspect of overall well-being. However, some research suggests a more complex relationship between body dissatisfaction and life satisfaction. In their study, Tiggemann and Slater [51] examined the impact of social media and Internet use on body satisfaction among adolescent girls. They found that exposure to idealized body images on platforms such as Facebook negatively influenced body satisfaction and ultimately contributed to lower overall life satisfaction among participants [51]. Given this complex relationship, promoting a healthy body image and fostering self-acceptance are essential for enhancing individuals' life satisfaction. Efforts to reduce body dissatisfaction through education, positive media portrayals, and self-acceptance initiatives can play a crucial role in improving overall psychological well-being and life satisfaction.

A negative correlation between body dissatisfaction and mood was observed in both vegetarians and nonvegetarians. This finding suggests that a negative body perception may be linked to an adverse emotional state, regardless of dietary pattern. This result aligns with previous studies, which have shown that body dissatisfaction is often associated with symptoms of depression and anxiety, as it negatively impacts self-esteem and psychological well-being [52]. Furthermore, recent research suggests that this phenomenon may be more pronounced in sociocultural contexts that emphasize unrealistic body standards, affecting both vegetarians and nonvegetarians [53]. However, the fact that this relationship is independent of diet type suggests that other factors, such as social pressure, self-image, or personal experiences, may play a mediating role. This highlights the need for interventions aimed at promoting body acceptance and improving emotional well-being, considering diet as a potential contextual factor rather than a determinant. The interplay between diet, body image, and mood warrants further exploration in future research to clarify underlying mechanisms and examine potential cultural or gender differences.

Finally, regardless of dietary pattern, a positive correlation was observed between body dissatisfaction and BMI, indicating that individuals with higher levels of body dissatisfaction tend to have a higher BMI. This finding aligns with existing evidence suggesting a close relationship between body weight and body image perception. Research indicates that elevated BMI is often associated with greater body dissatisfaction, likely because of societal pressures that promote thin ideals and stigmatize overweight individuals [54]. This phenomenon appears to be independent of diet type, as both vegetarians and nonvegetarians are influenced by the same cultural norms. However, some studies suggest that vegetarians may experience greater body image concerns because of ethical or health motivations behind adopting this diet, although findings on this matter remain inconsistent [55]. In addition, an elevated BMI may serve as a risk factor for negative body perception, further reinforced by experiences of weight-based discrimination and health-related concerns [50]. This finding highlights the importance of interventions aimed at promoting a healthy self-image while reducing weight-related stigma, regardless of dietary choices.

### 4.1. Limitations and Future Perspectives

One of the main limitations of this study is the use of accidental, nonprobability sampling, which introduces selection bias and may limit the representativeness of the sample, thereby affecting the generalizability of the results. In addition, another significant limitation arises from the reliance on self-report instruments to measure nutritional knowledge, physical activity, mood, body satisfaction, and life satisfaction. While these instruments are validated, biases inherent to self-reported data, such as the tendency to respond in a socially desirable manner or the overestimation/underestimation of certain behaviors or emotions, may impact the accuracy of the findings. Likewise, the cross-sectional design of this study, in which data were collected at a single point in time, limits the ability to establish causal relationships. While this design allows for the observation of associations between variables, it does not enable us to determine whether changes in nutritional knowledge, physical activity, or other factors directly influence mood, body satisfaction, or life satisfaction. To overcome this limitation, future studies could adopt a longitudinal design to examine causal relationships and assess how interventions impact well-being over time.

Likewise, the cross-sectional design of this study, in which data were collected at a single point in time, limits the ability to establish causal relationships. While this design allows for the observation of associations between variables, it does not enable us to determine whether changes in nutritional knowledge, physical activity, or other factors directly influence mood, body satisfaction, or life satisfaction. To overcome this limitation, future studies could adopt a longitudinal design to examine causal relationships and assess how interventions impact well-being over time. Increasing the sample size in future studies would enable more robust analyses with greater precision. Lastly, although this study differentiates between vegetarians and nonvegetarians, it does not explore the specific dietary patterns within each group, such as diet quality or the intake of key nutrients that may influence health and psychological well-being. The lack of a detailed assessment of dietary quality limits the ability to draw conclusions about how specific dietary practices influence well-being. Future studies would benefit from incorporating more comprehensive assessments of dietary patterns and nutritional quality to gain a more accurate understanding of their impact on mental and physical health.

### 4.2. Practical Implications and Public Health Relevance

The findings of this study offer several important considerations for nutrition education and lifestyle change programs targeted at both vegetarians and nonvegetarians. First, the significant associations observed between nutritional knowledge and dietary patterns, particularly in vegetarians, suggest that targeted educational interventions focusing on increasing nutritional awareness could be beneficial in promoting healthier eating habits. For vegetarians, this might involve reinforcing knowledge about key nutrients and dietary choices that ensure a balanced diet, while for nonvegetarians, education might focus on reducing dietary dissatisfaction and encouraging more plant-based consumption to improve body satisfaction and overall well-being. The strong association between body satisfaction and dietary patterns, particularly among nonvegetarians, underscores the need for tailored interventions that address both physical and psychological well-being.

In terms of public health, these findings highlight the importance of incorporating psychological elements, such as body satisfaction and mood, into nutrition and lifestyle education programs. Given that emotional well-being and dietary choices are closely intertwined, a more holistic approach that integrates these factors could enhance the effectiveness of public health campaigns aimed at improving dietary habits and promoting a healthier lifestyle across populations. These insights are especially relevant in Colombia, where there may be unique cultural and dietary influences that shape the relationship between nutrition, physical activity, and psychological health.

Although several of the correlations reported in this study, such as the relationship between body satisfaction and life satisfaction in vegetarians (*ρ* = −0.249), were statistically significant, they are described as “low” or “very low.” It is essential to interpret these weak correlations in terms of their practical relevance. While these associations may not indicate strong relationships, they nonetheless suggest meaningful patterns that warrant attention, particularly in the context of a multifaceted topic like nutrition and well-being. Small effect sizes are not uncommon in behavioral and psychological research, and even modest associations can have practical significance when considered alongside other factors.

For instance, the observed correlation between body satisfaction and life satisfaction, though weak, may still point to a broader relationship between self-perception and overall quality of life, especially in specific subgroups such as vegetarians. These findings could inform future interventions by encouraging a focus on small, yet potentially impactful, changes in diet and self-perception. Moreover, recognizing these correlations, however small, can provide a foundation for more in-depth investigations and longitudinal studies to better understand the nuances of these relationships over time.

## 5. Conclusion

This study observed significant associations between nutritional knowledge and dietary patterns, with a stronger relationship in vegetarians. In addition, there was a significant association between body satisfaction and dietary patterns, showing greater dissatisfaction in nonvegetarians. Negative correlations were found between body dissatisfaction and life satisfaction and mood in both vegetarians and nonvegetarians, as well as a positive correlation with BMI. In vegetarians, positive correlations were observed between mood and physical activity and BMI; besides, BMI was negatively correlated with nutritional knowledge. In nonvegetarians, nutritional knowledge showed positive correlation with life satisfaction.

While the cross-sectional design limits the ability to infer causality, the novelty of this study lies in its exploration of these relationships within a Colombian context, a population that has been understudied in this area. These findings provide valuable insights into the unique dietary behaviors and psychological well-being of Colombian adults, highlighting population-specific patterns that may differ from those observed in other cultural contexts. This research underscores the importance of nutritional education and the need for a comprehensive approach that includes dietary habits, physical activity, and emotional well-being to promote healthy habits and a better quality of life.

## Figures and Tables

**Table 1:** Sociodemographic characteristics of vegetarian and nonvegetarian participants.

Variables	Vegetarian (*n* = 157)		Nonvegetarian (*n* = 321)		Total	
	*n*	%	*n*	%	*n*	%
Age						
17–25	44	28.0	41	12.8	85	17.8
26–34	48	30.6	123	38.3	171	35.8
35–43	36	22.9	80	24.9	116	24.3
> 44	29	18.5	77	24.0	106	22.2
Sex						
Female	107	68.2	215	67.0	322	67.4
Male	50	31.8	106	33.0	156	32.6
Marital status						
Single	84	53.5	182	56.7	266	55.6
Married or with a partner	73	46.5	139	43.3	212	44.4
Socioeconomic status						
Status 1	23	14.6	40	12.5	63	13.2
Status 2	46	29.3	89	27.7	135	28.2
Status 3	56	35.7	119	37.1	175	36.6
Status 4	19	12.1	53	16.5	72	15.1
Status 5	10	6.4	17	5.3	27	5.6
Status 6	3	1.9	3	0.9	6	1.3
Educational level						
Technician	39	24.8	78	24.3	117	24.5
University	86	54.8	170	53.0	256	53.6
Postgraduate	32	20.4	73	22.7	105	22.0
Body mass index						
Underweight	7	4.5	5	1.6	12	2.5
Normal	116	73.9	164	51.1	280	58.6
Excess weight	34	21.7	152	47.4	186	38.9

**Table 2:** Mean differences between vegetarians and nonvegetarians.

Variables	Vegetarian (*n* = 157)		Nonvegetarian (*n* = 321)		*t*	*p*	*D*
	M	SD	M	SD			
Physical activity	3242.3	4128.8	2874.6	3421.1	1.03	0.304	0.10
Body dissatisfaction	16.27	7.40	18.64	8.19	−3.07	0.002	0.30
Life satisfaction	17.64	4.67	17.97	4.63	−0.71	0.477	0.07
Nutritional knowledge	15.13	2.56	13.61	2.95	5.51	0.000	0.55
Mood	66.90	15.83	65.29	13.95	1.13	0.258	0.11
BMI	23.34	4.93	25.16	3.99	−4.34	0.000	0.41

*Note:* M: mean.

Abbreviations: BMI, body mass index; SD, standard deviation.

**Table 3:** Correlations between variables with confidence intervals.

Vegetarians (*n* = 157)	1	2	3	4	5
1. Physical activity					
2. Body dissatisfaction	−0.06 [−0.22, 0.10]				
3. Life satisfaction	0.06 [−0.10, 0.22]	−0.26⁣^∗∗^ [−0.40, −0.10]			
4. Nutritional knowledge	0.05 [−0.11, 0.21]	−0.01 [−0.16, 0.15]	0.14 [−0.02, 0.29]		
5. Mood	0.21⁣^∗∗^ [0.05, 0.35]	−0.28⁣^∗∗^ [−0.42, −0.13]	0.54⁣^∗∗^ [0.42, 0.64]	−0.02 [−0.17, 0.14]	
6. Body mass index (BMI)	−0.04 [−0.20, 0.11]	0.30⁣^∗∗^ [0.15, 0.43]	0.03 [−0.13, 0.19]	−0.17⁣^∗^ [−0.32, −0.01]	0.16⁣^∗^ [0.00, 0.31]

**Nonvegetarians (*n* = 321)**	**1**	**2**	**3**	**4**	**5**

1. Physical activity					
2. Body dissatisfaction	0.02 [−0.09, 0.13]				
3. Life satisfaction	0.04 [−0.07, 0.14]	−0.29⁣^∗∗^ [−0.38, −0.18]			
4. Nutritional knowledge	−0.01 [−0.12, 0.10]	0.02 [−0.09, 0.13]	0.14⁣^∗^ [0.03, 0.25]		
5. Mood	0.01 [−0.09, 0.12]	−0.36⁣^∗∗^ [−0.45, −0.26]	0.42⁣^∗∗^ [0.32, 0.51]	0.04 [−0.07, 0.15]	
6. Body Mass index (BMI)	0.05 [−0.06, 0.16]	0.29⁣^∗∗^ [0.18, 0.38]	−0.12⁣^∗^ [−0.23, −0.01]	−0.00 [−0.11, 0.11]	0.03 [−0.08, 0.14]

*Note:* M and SD are used to represent mean and standard deviation, respectively. Values in square brackets indicate the 95% confidence interval for each correlation. The confidence interval is a plausible range of population correlations that could have caused the sample correlation (Cumming, 2014).

⁣^∗^*p* < 0.05.

⁣^∗∗^*p* < 0.01.

## Data Availability

The data that support the findings of this study are available on request from the corresponding author. The data are not publicly available because of privacy or ethical restrictions.
